# Exosomes in osteoarthritis: Updated insights on pathogenesis, diagnosis, and treatment

**DOI:** 10.3389/fcell.2022.949690

**Published:** 2022-07-26

**Authors:** Wen-Jin Fan, Di Liu, Lin-Yuan Pan, Wei-Yang Wang, Yi-Lan Ding, Yue-Yao Zhang, Rui-Xi Ye, Yang Zhou, Sen-Bo An, Wen-Feng Xiao

**Affiliations:** ^1^ Xiangya School of Medicine, Central South University, Changsha, China; ^2^ Department of Orthopedics, Xiangya Hospital, Central South University, Changsha, China; ^3^ Department of Clinical Nursing, Xiangya Hospital, Central South University, Changsha, China; ^4^ Department of Orthopaedics, Shandong Provincial Hospital Affiliated to Shandong First Medical University, Jinan, China; ^5^ National Clinical Research Center for Geriatric Disorders, Xiangya Hospital, Central South University, Changsha, China

**Keywords:** exosomes, osteoarthritis, biomarkers, cartilage, cartilage tissue engineering

## Abstract

Osteoarthritis (OA) has remained a prevalent public health problem worldwide over the past decades. OA is a global challenge because its specific pathogenesis is unclear, and no effective disease-modifying drugs are currently available. Exosomes are small and single-membrane vesicles secreted via the formation of endocytic vesicles and multivesicular bodies (MVBs), which are eventually released when MVBs fuse with the plasma membrane. Exosomes contain various integral surface proteins derived from cells, intercellular proteins, DNAs, RNAs, amino acids, and metabolites. By transferring complex constituents and promoting macrophages to generate chemokines and proinflammatory cytokines, exosomes function in pathophysiological processes in OA, including local inflammation, cartilage calcification and degradation of osteoarthritic joints. Exosomes are also detected in synovial fluid and plasma, and their levels continuously change with OA progression. Thus, exosomes, specifically exosomal miRNAs and lncRNAs, potentially represent multicomponent diagnostic biomarkers for OA. Exosomes derived from various types of mesenchymal stem cells and other cell or tissue types affect angiogenesis, inflammation, and bone remodeling. These exosomes exhibit promising capabilities to restore OA cartilage, attenuate inflammation, and balance cartilage matrix formation and degradation, thus demonstrating therapeutic potential in OA. In combination with biocompatible and highly adhesive materials, such as hydrogels and cryogels, exosomes may facilitate cartilage tissue engineering therapies for OA. Based on numerous recent studies, we summarized the latent mechanisms and clinical value of exosomes in OA in this review.

## Introduction

Osteoarthritis (OA) remains a leading joint disease in the aging population as well as the major cause of chronic pain and disability worldwide (Helmick et al., 2008; Hunter et al., 2014; Prieto-Alhambra et al., 2014). Whole joint structures deteriorate in this degenerative articular disorder, including synovium, ligaments, subchondral bone, articular cartilage and periarticular muscles (Brandt et al., 2006). Although the local cartilage damage is the fundamental pathological change of knee OA, the pathogenesis and epidemiology of OA are not completely clear, but multifactorial genetic, biological, and biomechanical factors are implicated. Current conservative treatments can only temporarily relieve osteoarthritic symptoms and partially delay the progression of OA (Crawford et al., 2013), and effective disease-modifying drugs are not available. Total knee arthroplasty (TKA) is a relatively effective treatment modality targeting advanced OA in clinical practice. However, the lifespan of prostheses is limited, and patient quality of life is inevitably affected (Kester et al., 2016). Therefore, studies are needed to further investigate definite mechanisms and effective therapeutic strategies.

Exosomes are extracellular vesicles (EVs) secreted by ubiquitous cells and carry various cellular constituents (Kalluri and LeBleu, 2020). Once released, exosomes are capable of remodeling the extracellular matrix and transmitting signaling molecules via the transport of biological cargo and the accumulation of specific cellular components. The diagnostic and therapeutic applications of exosomes in various diseases involving cardiovascular diseases, neurodegenerative diseases and cancer, are mainly attributed to their bioactive contents (Kalluri and LeBleu, 2020). Moreover, the inner cargo constituents of exosomes are available by means of biological fluid (liquid biopsy) sampling, which highlights their potential roles in the diagnosis of various diseases and in determining patient prognosis (Kalluri and LeBleu, 2020).

Exosomes have been detected in osteoarthritic synovial fluid and have emerged to play pivotal roles in the pathogenesis and therapy of OA (György et al., 2012; Headland et al., 2015). Different types of mesenchymal stem cell (MSC)-derived exosomes potentially serve as a novel approach for OA treatment based on the potent proliferation and differentiation abilities of multipotent stem cells (Asghar et al., 2020). Recent studies have demonstrated that MSC-derived exosomes alleviate knee OA and temporomandibular joint OA by alleviating inflammation and restoring matrix homeostasis (Zhang et al., 2019; Li et al., 2020). Various MSC-based therapies are in clinical trials for tissue regeneration of bone and cartilage, and exosomes derived from bone mesenchymal stem cells (BMSCs), synovial mesenchymal stem cells (SMSCs), adipose tissue mesenchymal stem cells (AMSCs), human embryonic stem cells (hESCs) and other stem cells may also function as potential therapeutic targets for OA (Ni et al., 2020). Additionally, the content of exosomes also dynamically varies during OA progression (Ruivo et al., 2017; Tai et al., 2018). Specifically, microRNAs (miRNAs), long noncoding RNAs (lncRNAs) and circular RNAs (circRNAs) can be transferred from MSCs to osteoarthritic cells through exosomes to treat OA. However, these studies are still in the primary research stage; thus, exosomes have not been applied in the clinical treatment of OA. MSC-Exos, as a new engineering technology, are gradually becoming a promising strategy for OA (Ni et al., 2020). In this review, we further shed light on the underlying biofunctions and therapeutic potential of exosomes in OA.

## Exosomes

### Biogenesis of exosomes

The biogenic process of exosome formation is initiated by a double invagination of the plasma membrane. Then, multivesicular bodies (MVBs) carrying intraluminal vesicles (ILVs) form and fuse with the cell membrane and are ultimately secreted as exosomes through exocytosis (Kalluri and LeBleu, 2020). Extracellular constituents and integral proteins on the cell surface access cells by means of endocytosis and invagination of the cell membrane to form early-sorting endosomes (ESEs). Subsequently, the ESEs combine with the endoplasmic reticulum (ER) and trans-Golgi network (TGN) or the constituents of the ER and TGN. Therefore, some ESEs serve as carriers of membrane and luminal constituents and subsequently form late-sorting endosomes (LSEs) (Kalluri, 2016; Hessvik and Llorente, 2018; van Niel et al., 2018; Willms et al., 2018; Mathieu et al., 2019; McAndrews and Kalluri, 2019). The ILVs are generated by the second invagination, and the cargo of exosomes is also modified simultaneously. Eventually, with the specific accumulation of ILVs, MVBs are either degraded by the lysosomes or transported to the cell membrane, contributing to the secretion of exosomes with a lipid bilayer orientation comparable to that of the cell membrane (Kahlert and Kalluri, 2013; van Niel et al., 2018). Various other specific vesicular activities within cells and other influencing factors, such as autophagy, culture systems, lysosomal pathways, Golgi apparatus–derived vesicle trafficking cell types, and genomic conditions, may affect the biogenic process as well (Mathieu et al., 2019).

### Biofunctions of exosomes

Studies have demonstrated that exosomes exert vital functions in numerous biological activities, including angiogenesis, apoptosis, antigen presentation, intercellular signaling and inflammation, partially by utilizing the capability to transport RNA, enzymes, proteins, and lipids. Thus, exosomes influence the physiology and pathology of different diseases, such as atherosclerosis, diabetes-related cardiovascular disease, breast and pancreatic cancers, neurodegenerative diseases and OA (Gurunathan et al., 2019). The biofunctions of exosomes are mainly attributed to the inner composition and the phenotypical and molecular changes they exert on recipient cells via intercellular communication (Kalluri and LeBleu, 2020). For example, exosomes carrying tumor-specific RNAs, such as circulating miR-141 and miR-375, can be utilized for the diagnosis of prostate cancer (Mitchell et al., 2008; Silva et al., 2011). Serum exosomes loaded with proteoglycan glypican-1 (GP1) can serve as diagnostic markers for pancreatic cancer (Melo et al., 2015). Plasma exosomes isolated from the pleural effusions of lung cancer patients contain higher levels of CD151, CD171, and tetraspanin8, which represent promising biomarkers for lung cancer diagnosis (Li et al., 2021b). In addition, emerging studies have reported that by incorporating small molecules or nucleic acid drugs into exosomes and subsequently transporting them to specific types of cells or tissue, exosomes can be engineered for targeted drug delivery, providing new insights for cell-free therapies for a variety of diseases (Liang et al., 2021). However, the most significant role of exosomes involves use as a “vehicle” for intercellular communications, in which cargo contents are transferred to recipient cells (Lee et al., 2020). The cargo composition of exosomes is dependent on parent cells, however, regardless of the cell origin, exosomes carry some common components, such as heat shock proteins, tetraspanin, nucleic acids, membrane transportation and fusion proteins, biosynthesis-related proteins and lipids (Colombo et al., 2014; Choi et al., 2015). The biofunctions of exosomes in intercellular signaling can be made more complex due to the specific mechanisms and pathways involved in exosome uptake and the specific putative mechanisms of exosomes derived from distinct cell types. (Mulcahy et al., 2014; Mathieu et al., 2019).

In addition, exosomes play essential roles in inflammatory processes. Ridder et al. (Ridder et al., 2014) found that exosomes delivered mRNAs to recipient cells, and the process is facilitated by the stimulation of immune cells that produced exosomes of severe inflammation (peritonitis) or chronic inflammation (subcutaneous tumor). In another study, exosomes suppressed the immune response through the transfer of miRNAs, presenting of molecules to regulate the immune response, including programmed death-ligand 1 (PD-L1) and Fas ligand (FasL), on their surface (Chen et al., 2018). Moreover, circulating exosomes stimulate the release of proinflammatory cytokines, such as interleukin 6 (IL-6), tumor necrosis factor alpha (TNFα), granulocyte colony-stimulating factors, and chemokine C-C motif ligand 2 (CCL2) (Chow et al., 2014).

Exosomes also play vital roles in angiogenesis, which is associated with differentiation, neovascularization, promoted renewal of blood flow, and capillary network construction by loading a spectrum of miRNAs, including miR210, miR126, miR132, and miR21, from MSCs (Chen et al., 2010; Ma et al., 2018). In addition, exosomes also participate in apoptosis. Dying cells release apoptotic bodies as products of apoptotic cell disassembly, and the apoptotic bodies finally become one of the major types of EVs (Caruso and Poon, 2018). Sun et al. (2019) found that miR-664-5p transferred through BMSC-derived exosomes impaired the apoptotic death of ovarian granulosa cells by targeting p53. Tao et al. (2017a) adopted a dexamethasone (DEX)-treated cell model *in vitro* and methylprednisolone (MPS)-treated *in vivo* rat model and drew the conclusion that exosomes derived from human platelet-rich plasma prevented apoptosis through the AKT/BAD/BCL-2 signaling pathway.

Based on the abundant biofunctions of exosomes, exosomes may be applied in new and innovative approaches for the diagnosis and treatment of OA.

### Exosomes in various diseases

Studies have confirmed that exosomes function in the emergence of metabolic diseases and cardiovascular health. Cancer cell-derived exosomes functionally induced the progression of cachexia and paraneoplastic syndrome by changing the metabolism of normal cells, including adipocytes and pancreatic islet cells. Platelet-derived exosomes function to reduce the expression level of the macrophage scavenger receptor CD36, thus reducing the intake of harmful cholesterol to influence atherosclerosis (Srikanthan et al., 2014). In addition, exosomes are related to some symbolic characteristics of cancer, which influence neoplasia, metastasis, paraneoplastic syndromes, tumor growth, and resistance to therapy (Hanahan and Weinberg, 2011). Moreover, exosomes may participate in detoxification and neuroprotective processes in neurodegenerative diseases by affecting the collection of unfolded and abnormally folded proteins in the brain and play roles in the clearance of misfolded proteins. Intercellular communication through exosomes including α-synuclein, amyloid β, and prions, leads to inflammatory signaling in Parkinson’s, Alzheimer’s, and Creutzfeldt-Jacob diseases (Li and Barres, 2018). In recent years, the emerging use of MSC-based therapies for OA also contributes to the paracrine secretion of nutritional factors (Toh et al., 2014), especially exosomes. Exosomes derived from MSCs safeguard bone and cartilage against damage in OA by enhancing the expression level of chondrocyte markers including type II collagen and aggrecan; inhibiting inflammatory markers (iNOS); suppressing apoptosis of chondrocytes; stimulating chondrocyte migration and proliferation (Zhu et al., 2017); and inhibiting the activation of macrophages (Cosenza et al., 2017). Therefore, exosomes may participate in the pathogenesis and progression of various diseases including OA.

## Exosomes in OA pathogenesis

Exosomes play an essential role in biological processes in OA chondrocytes and relevant inflammatory cells (Tkach and Théry, 2016). Previous studies have revealed that T lymphocytes and macrophages are major inflammatory cells involved in OA (de Lange-Brokaar et al., 2012). Synovial macrophages are mainly located in the synovium lining layer and are stimulated in inflammatory conditions (Haywood et al., 2003). By releasing proinflammatory cytokines, growth factors and enzymes, macrophages participate in the stimulation of angiogenesis, recruitment of leukocytes and lymphocytes, fibroblast proliferation, and protease secretion, thus resulting in joint destruction (Griffin and Scanzello, 2019). Domenis et al. (2017) revealed that synovial fibroblast (SF)-derived exosomes from OA patients facilitated macrophages to generate an array of chemokines and proinflammatory cytokines, such as IL-1β (interleukin-1 beta), CCL (chemokine ligand) 8 (CCL8), CCL15 and CCL20, whereas the release of IL-16, IL-8 and CCL7 was downregulated, resulting in cartilage destruction and local inflammation in joints. However, the underlying mechanisms of how SF-derived exosomes facilitated macrophages to produce these chemokines have not been explored but require further study. Significantly, IL-1β, as an upstream cytokine involved in the local inflammation of OA, enhances the expression and secretion level of other proinflammatory cytokines, such as IL-8 and IL-6, and the production of proteases, mainly matrix metalloproteinases (MMPs). Furthermore, exosomes derived from IL-1β-induced SFB stimulated angiogenic activities, including migration and tube formation of human umbilical vein endothelial cells, are likely involved in the expression of OA-related genes stimulated by exosomes. This process is associated with the increased angiogenesis in osteoarthritic joints. However, the specific pathways involved are unclear (Kato et al., 2014). Moreover, M1 macrophages stimulated by exosomes derived from SF lead to the secretion of MMP12 and MMP7 and the suppression of MMP8 synthesis thus contributing to the degradation of extracellular matrix, in which exosomes may function via specific and alternative pathways. Nevertheless, the specific pathways involved should be further explored (Domenis et al., 2017). Therefore, via the stimulation of M1 macrophages to secrete critical molecules functioning in the inflammatory process and cartilage damage, SF-derived exosomes of inflammatory joints of patients with OA can consequently participate in disease development and trigger and contribute to the extension of the inflammatory process (Buzas et al., 2014; Malda et al., 2016).

Exosomes modulate the biological functions of osteoarthritic cells by transferring miRNAs and lncRNAs as well (Xie et al., 2020). Wu et al. (2019) used a destabilization of the medial meniscus (DMM)-induced OA mouse model and demonstrated that exosomes derived from infrapatellar fat pad (IPFP) MSCs protected articular cartilage from degeneration through relatively complex mechanisms in which exosomal miR-100-5p-modulated suppression of the mTOR-autophagy pathway is involved. In another study, Mao et al. (2018) showed that cartilage development and degradation were mediated by exosomal miR-92a-3p through the inhibition of WNT Family Member 5A (WNT5a) in chondrogenesis and pathogenesis of OA. Yan et al. (2021) utilized an SD rat cartilage defect model *in vivo* in the experiment and revealed that umbilical cord mesenchymal stem cell (UMSC)-derived exosomal lncRNA H19 promoted the migration of chondrocytes and matrix secretion, and suppressed apoptosis and senescence by acting as a competing endogenous RNA (ceRNA) against miR-29b-3p to increase FoxO3 in chondrocytes. Consequently, given variations in the levels of exosomal miRNAs, these miRNAs have potential to serve as biomarkers for OA diagnosis. In addition, exosomal miRNAs and lncRNAs play a significant role in improving osteochondral activities, demonstrating their therapeutic potential in OA.

Exosomes also contribute to the abnormal calcification and destruction of cartilage in OA. Abnormal calcification of cartilage is a significant pathological change of temporomandibular joint (TMJ) OA. Liu et al. (2022a) explored the connection between chondrocyte-derived exosomes and cartilage calcification and demonstrated that the secretion of exosomes in abnormal TMJ enhanced calcification in degenerative cartilage noted in TMJ OA.

Exosomes may serve as a novel regulatory strategy in OA pathogenesis via excessive catabolism and promotion of chondrocyte apoptosis (Kato et al., 2014). A recent study has found that by downregulating autophagy and p21 expression, vascular endothelial cell-derived exosomes restrained the capability of chondrocytes to defend against oxidative stress, thus increasing the cellular ROS content and contributing to apoptosis. These results indicate that vascular endothelial cell-derived exosomes facilitate OA pathogenesis and promote OA progression (Yang et al., 2021).

## Clinical applications of exosomes in OA

### Diagnostic potential of exosomes in OA

For the purpose of controlling the development of OA in a timely and effective manner, early-term diagnosis of OA is particularly important (Bhimani et al., 2018). Several biochemical markers for OA diagnosis have been proposed (Glyn-Jones et al., 2015). Currently, to determine the severity and development of OA, researchers mainly concentrate on biomarkers in plasma and synovial fluid that are associated with inflammatory and angiogenetic factors (Lipina et al., 2017). As novel biomarkers, exosomes are expected to be utilitarian as they carry distinct information from secreted cells, including miRNAs and lncRNAs. The expression of some molecules, including both exosomes and exosomal lncRNAs in the blood, is not altered in OA, indicating that OA is a local lesion and is limited to joint synovial fluid (Zhao and Xu, 2018). However, although the production of exosomes in synovial fluid is obviously increased in patients with OA compared with healthy people, no obvious distinction is observed between early-stage OA and advanced OA. To identify osteoarthritic stages of OA, Zhao et al. (Zhao and Xu, 2018) demonstrated that the expression level of synovial fluid-derived exosomal lncRNAs was increased in the primary stage of OA, indicating that lncRNAs are the underlying biomarkers for OA. Exosomal lncRNA prostate-specific transcript 1 (PCGEM1) showed appreciable differences in different stages of OA and was significantly elevated in advanced OA compared with primary OA as well as advanced OA compared with healthy controls, suggesting that lncRNA PCGEM1 from synovial fluid may represent a potent marker for identifying primary OA and advanced OA. In addition, still some studies have revealed that the expression of some exosomal miRNAs is modified in OA patients. Meng et al. (2018) showed that plasma exosomal miR-193b expression was obviously reduced in patients with OA compared with normal controls. In addition, circRNAs represent another family of noncoding RNAs with a closed ring structure in the cytoplasm or exosomes (Li et al., 2015). Studies have increasingly revealed that various circRNAs affect OA progression by interacting with cirRNA/miRNA/mRNA pathways, thereby influencing homeostasis. Therefore, circRNAs including hsa-circ-003213 in peripheral blood and hsa-circ-0104595 in synovial fluid, have the potential to be transported via bodily fluids and could serve as diagnostic biomarkers (Zhang et al., 2021b; Mao et al., 2021) (Table 1).

**TABLE 1:** Exosomes as biomarkers in OA.

Exosomes	Biomarkers	Expression level	References
Synovial fluid-derived exosomes	lncRNA PCGEM1	Upregulated	Zhao and Xu, (2018)
Plasma-derived exosomes	miR-193b	Downregulated	Meng et al. (2018)
Stem cell-derived exosomes	Hsa-circ-0104595	Upregulated	(Zhang et al., 2021b; Mao et al., 2021)

### Therapeutic value of exosomes in OA

#### Exosomes and cartilage tissue repair

Cartilage is a specialized type of connective tissue consisting of collagen fibers, hyaluronic acid, proteoglycans and chondrocytes (Carballo et al., 2017). Effectual remodeling of impaired cartilage tissues is often limited by the typical avascular structure and restriction in interchanges of signaling molecules, adequate provision of oxygen and nutrients, and penetration of precursor cells (Krishnan and Grodzinsky, 2018). OA induces pathophysiological destruction of the cartilage tissue architecture. After the flaking and cracking of cartilage tissues at OA early stages, the matrix becomes calcified and expands. The matrix ultimately replaces the articular region in the local cartilage tissue, resulting in delamination and the exposure of subchondral bone tissues. Different paracrine signaling pathways in MSC populations induce cartilage tissue regeneration, in which exosomes act as important signaling messengers for intercellular communication and stimulation for cartilage tissue repair (Kim et al., 2020). However, the exact mechanism of physiological responses mediated by exosomes is complicated and involves the induction of chondrocyte proliferation, suppression of the apoptosis of cells in cartilage, downregulation of the levels of proinflammatory synovial cytokines, increased infiltration of M2 macrophages and the regulation of the immune response (Zhang et al., 2018).

#### Stem cell-derived exosomes in OA

##### Bone mesenchymal stem cell-derived exosomes

BMSC-Exos accelerate a series of pathophysiological processes, such as angiogenesis and osteogenesis (Zhang et al., 2020). BMSC-Exos considerably facilitate the repair of destroyed cartilage and subchondral bone (Asghar et al., 2020). In a study, Cosenza et al. (Cosenza et al., 2017) revealed that BMSC-Exos upregulated the expression of aggrecan and type II collagen while decreasing the expression levels of MMP-13, a disintegrin and metalloproteinase with thrombospondin motifs 5 (ADAMTS5) and iNOS. Moreover, exosomes from BMSCs treated with TGFβ3 markedly upregulated the expression levels of anabolic marker genes (ACAN, COL1, and COL2B) and downregulated the levels of catabolic and inflammatory marker genes (MMP-13, ADAMTS5, and iNOS) in osteoarthritic chondrocytes (Cosenza et al., 2017). BMSC exosomes also protect chondrocytes from IL-1β-induced apoptosis through the p38, ERK, and AKT pathways (Qi et al., 2019). In addition, chondrocyte proliferation and migration are also promoted by BMSC-Exos. One of the underlying mechanisms is that BMSC-Exos reversed the reduction of collagen type II, SOX9, aggrecanases, and proteoglycan four expression levels induced by OA (Zhou et al., 2020; Xu and Xu, 2021). Furthermore, BMSC-Exos modulate the activities of synovial fibroblasts and macrophages. PTGS2 is one of the target genes of miR-26a-5p, which is significantly upregulated in OA, thus inhibiting PTGS2 and exert a protective effect on OA. BMSC-derived exosomes deliver miR-26a-5p into synovial fibroblasts thus attenuating the impairment of synovial fibroblasts potentially by targeting PTGS2 (Jin et al., 2020) (Fukai et al., 2012). In addition, Raghu et al. (van den Bosch et al., 2016; Raghu et al., 2017) found that monocytes and microphage enhanced local inflammation and tissue damage in OA. BM-MSCs suppress the inflammation of the synovium by inducing the polarization of macrophages toward an anti-inflammatory phenotype (Schelbergen et al., 2014). This process is mediated by PGE2 detected in BMSC-Exos. Current studies have indicated that the production and contents of exosomes are regulated through the drug intervention or gene modification, which may alter the BMSC-Exo cellular effect on targeted cells. Thus exosomes may function as an appropriate therapeutic approach for OA (Gurunathan et al., 2019).

##### Adipose mesenchymal stem cell-derived exosomes

AMSCs are efficient in regenerating cartilage and regulating inflammatory reactions and are thus considered a preferable cell source for OA treatment (Lee et al., 2019). By modulating the local environment to make cartilage remodeling more advantageous via the paracrine secretion of nutritious molecules, AMSCs inhibit cartilage erosion and enhance joint function (Damia et al., 2018).

Tofiño-Vian et al. (2017) found that the paracrine effects of AMSCs were mediated by exosomes on osteoarthritic osteoblasts. Furthermore, AMSC-derived exosomes impaired the production of proinflammatory mediators in osteoarthritic chondrocytes and increased the levels of anti-inflammatory cytokines IL-10 and collagen II, which are specific to chondrocytes, among which the exosomal component annexin A1 may participate in the anti-inflammatory process (Tofiño-Vian et al., 2018). These results suggest that exosomes derived from AMSCs may become a prospective therapeutic modality for OA.

##### Synovial mesenchymal stem cell-derived exosomes

SMSCs are a type of articular MSCs found in the synovial fluid filling the joint cavity (Jo et al., 2014; Katare et al., 2014; Lee et al., 2015). SMSCs exhibit an increased ability to differentiate into chondrocytes, but a reduced ability for adipogenic, osteogenic and neurogenic differentiation than other types of MSCs (Xin et al., 2014; Burger et al., 2015). BMSCs and AMSCs have been used for the treatment of OA. However, as synovium and cartilage have the same origin during the development of synovial joints, synovial membrane-derived MSCs (SMMSCs) are especially suitable for cartilage (Archer et al., 2003; Koyama et al., 2008). Given advantageous chondrogenic differentiation capacity *in vitro* (Shirasawa et al., 2006; Kurth et al., 2007), transplantation of SMSCs has been used for OA treatment (Xu et al., 2021).

Studies by Koizumi et al. (Koizumi et al., 2016; Zhu et al., 2017) have shown that SMSC-derived exosomes significantly facilitated cartilage regeneration and suppressed OA development. Exploiting the WNT5a/WNT5b/YAP pathway, exosomes derived from SMSCs enhanced chondrocyte emigration and proliferations. Nevertheless, these SMSC exosomes inhibited the secretion of extracellular matrix (ECM). Tao et al. (2017b) used miR-140-5p to transfect SMSCs and derived exosomes from SMSCs or SMSC-140s. They found that exosomes treated with miR-140-5p (SMSC-140-Exos) facilitated chondrocyte proliferation and migrations without decreasing ECM secretion *in vitro*. Experiments in rat models demonstrated that SMMSC-derived exosomes modulated bone regeneration as well (Guo et al., 2016).

##### Embryonic mesenchymal stem cell-derived exosomes

Exosomes secreted by ESC-MSCs attenuate OA partially by regulating the equilibrium between the production and degeneration of cartilage matrix. Embryonic stem cell-induced mesenchymal stem cells (ESC-MSCs) enhanced Col II expression levels and decreased ADAMTS5 expression in the cartilage matrix (Wang et al., 2017). However, compared with traditional cellular therapies, MSC exosome therapies can be better controlled to reformulate and support different routes of administration. Weekly injection of human embryonic MSC exosomes into articular tissue promoted amelioration of critical-sized osteochondral damage and induced an orderly regeneration of cartilage and subchondral bone in an adult immunocompetent rat model (Zhang et al., 2016). Moreover, in the context of a lack of MHC class I/II proteins, human MSC exosomes can be applied in immunocompetent animals without the need for immunosuppression (Tan et al., 2014). Wang et al. (2017) also demonstrated that the development of cartilage damage in an OA model can be prevented by ESC-MSC-derived exosomes. This effect may be facilitated by direct contact and fusion between chondrocytes and exosomes, with a decrease in the matrix degradation enzyme ADAMTS5 and a balancing increase in the extracellular matrix protein collagen type II. Nevertheless, the mechanisms of EMSC-Exos in the treatment of OA need to be further explored.

##### Other stem cell-derived exosomes

Liu et al. (2022b) utilized an *in vitro* IL-1β-induced OA model and an *in vivo* rat KOA model and concluded that the proliferation and migration capacity of chondrocytes were promoted while apoptosis was effectively reduced after treatment with human urine-derived stem cell (hUSCs)-derived exosomes. Nevertheless, ECM degradation was also aggravated by destroying the constituents of ECM, including collagen II. However, after transfecting with miR-140 and increasing miR-140 expression, the proliferation and migration capability were further enhanced without destroying the ECM compared with hUSC-Exos. Therefore, intra-articular injection of hUSC-140-Exos has the potential to attenuate the development of early KOA and protect knee articular cartilage from further acute destruction through the regulation of ECM homeostasis and subchondral bone restoration.

Human induced pluripotent stem cells (iPSCs) are similar to embryonic stem cells in structure, self-regeneration, and specialization ability and are stimulated from somatic cells specific to patients (Kang et al., 2009; Hirschi et al., 2014). Through intra-articular administration of iPSCs-derived exosomes (iMSC-Exos) and SMMSC-Exos in a mouse model of OA induced by collagenase, Zhu et al. (Zhu et al., 2017) concluded that iMSC-Exos and SMMSC-Exos alleviated OA by stimulating chondrocyte movement and proliferation.

Compared with SMMSC-Exos, iMSC-Exos had superior therapeutic effects. iMSC-Exos probably function as a novel therapeutic target for OA because autologous iMSCs are theoretically inexhaustible, Compared with mature stem cells, such as BMSCs and AMSCs, amniotic fluid MSCs represent preferable exosome sources based on size and surface marker expression, and amniotic fluid MSCs are produced at higher levels from amniotic fluid cells (Tracy et al., 2019). Zavatti et al. (2020) conducted an experiment to study the effect of the secreted exosomes compared with their amniotic fluid stem cell (AFSC) source. These researchers utilized a MIA-induced animal model of osteoarthritis simulating a chronic and retrogressive process, where inflammation also participated and contributed to inversible joint degeneration and confirmed that by stimulating the movement and proliferation of repairing cells and enhancing the synthesis of cartilage matrix, AFSC-derived exosomes generated effective cartilage repair.

Yan et al. (Yan and Wu, 2020) used umbilical MSC (U-MSC) exosomes generated by typical two-dimensional (2D) tissue culture polystyrene flasks and 3D microgravity environment culture for the treatment of cartilage repair and demonstrated that the use of exosomes sourced from umbilical MSCs (U-MSC-Exos) also protected chondrocytes, activated cell proliferation, migration, and synthesis of the matrix, and reduced apoptosis. Moreover, exosomes derived from 3D-culture were more prolific and biologically active. In terms of sustaining the phenotypic stability of chondrocytes, 3D-Exos exhibited better capacity than 2D-Exos, which may partially contribute to a better capacity to promote the migration of chondrocytes and a prominent capacity in balancing matrix synthesis through the TGF-β1-dependent Smad2/3 signaling pathway. MSC-derived exosomes exhibit emerging potential as targets of especially acute examination, and several types of MSC derived exosomes have shown therapeutic potential in OA. However, comparisons between different MSC-derived exosomes are needed to optimize therapy utilizing MSC-derived exosomes (Tracy et al., 2019).

Above all, studies on exosomes derived from antler stem cells (ASCs) also provide new insights into novel therapeutic approaches for OA. Wang et al. (2019) detected genetic changes and gene expression in ruminant headgear and demonstrated that the great proliferation and differentiation potential of ASCs may be attributed to their origin from cranial neural crest cells. Lei et al. (2022) used an anterior cruciate ligament transection (ACLT) surgery-induced OA mouse model and found that ASC-derived exosomes attenuated human stem cell senescence and ameliorated cartilage degeneration. Consequently, ASC may represent an appropriate source for treatment based on exosomes.

#### Exosomes from other cells or tissues

The majority of current studies mainly concentrate on MSC-derived exosomes with respect to their diagnostic and therapeutic functions in OA. Nevertheless, exosomes derived from sources other than MSCs have therapeutic potential for OA. An *in vivo* study indicated that platelet-rich plasma (PRP)-derived exosomes increased proliferation and movement while decreasing apoptosis of osteoarthritic chondrocytes stimulated by IL-1β. Moreover, PRP-Exos activate the WNT/β-catenin signaling pathway and protect cartilage from degradation. Notably, the therapeutic outcome is even more satisfying than that of stimulated PRP (Liu et al., 2019).

Infrapatellar fat pad (IPFP)-derived exosomes have also exhibited potential utilization in the treatment of OA in DMM-induced OA models. Facilitated by exosomal miR-100-5p, which interacts with mammalian target of rapamycin (mTOR) and its downstream cofactors in chondrocytes, IPFP-Exos not only attenuated articular cartilage damage but also promoted gait function (Wu et al., 2019; Li et al., 2021a).

Zheng et al. (2019) demonstrated that exosomes derived from chondrocytes had effects on OA by regulating the metabolism of chondrocytes. They found that exosomes derived from chondrocytes delayed IL-1β-induced chondrocyte degeneration and restored the metabolism of damaged chondrocytes through the regeneration of the destroyed mitochondria via the supplementation of exosome proteins. Additionally, chondrocyte exosomes modulated the polarization of macrophages and increased the M2 phenotype to regulate immune reactivity in OA. An intracellular injection of exosomes also successfully mitigated the progression of OA, demonstrating the therapeutic potential of chondrocyte exosomes in an OA model.

Synovial fibroblast (SFC)-derived exosomes have also shown a great capability to reduce chondrocyte inflammation and cartilage destruction. An *in vivo* study by Zhou et al. revealed that SFCs-miRNA-126-3p-Exos maintained subchondral bone structure and suppressed synovial inflammation-mediated cartilage degeneration and articular cartilage chondrocyte apoptosis and inflammation in OA model rats (Zhou et al., 2021). These results indicate that SFC-miRNA-126-3p-Exos should be explored for therapeutic use in the treatment of OA.

Collectively, exosomes from stem cells including BMSCs, AMSCs, SMSCs, and ESCs, and other stem cells and other cells or tissues have shown powerful cartilage restoration ability and repressed OA progression in OA models. Still in the animal experiment stage, more research endeavors are desired to further clarify the specific mechanisms of exosomes in the treatment of OA and identify the most appropriate cell source (Table 2).

**TABLE 2:** Therapeutic effects of exosomes in OA.

Exosomes	Target cells	Mechanisms	Biological effects on OA	References
BMSCs-derived exosomes	Osteoarthritic chondrocytes	ERK, AKT and p38 pathways	Protect chondrocytes from apoptosis	(Fukai et al., 2012; Cosenza et al., 2017; Qi et al., 2019; Jin et al., 2020)
	Synovial fibroblasts	Upregulate the expression of anabolic markers genes and decrease catabolic and inflammatory markers genes	Facilitate the repair of injured cartilage and subchondral bone	
	Macrophages	MiR-26a-5p/PTGS2 pathway	Accelerate damage of synovial fibroblasts	
			Suppress activities of synovial fibroblasts and macrophages	
AMSCs-derived exosomes	Osteoarthritis chondrocytes	Upregulate cytokine IL-10 and collagen II and decrease proinflammatory mediators	Cartilage regeneration and inflammatory modulation	Tofiño-Vian et al. (2018)
SMSCs-derived exosomes	Articular chondrocytes	WNT5a/WNT5b/YAP way	Stimulate the proliferation and movement of chondrocytes	(Koizumi et al., 2016; Tao et al., 2017b; Zhu et al., 2017)
		MiR-140-5p mediated protection of ECM *in vitro*	Facilitate cartilage regeneration and suppress OA progression	
			Regulate bone remodeling	
EMSCs-derived exosomes	Osteoarthritic chondrocytes	Balance the synthesis and degradation of cartilage matrix	Prevent the development of cartilage destruction	(Tan et al., 2014; Zhang et al., 2016)
			Regeneration of cartilage and subchondral bone	
hUSCs-derived exosomes	Osteoarthritic chondrocytes	Decrease the expression of endothelial growth factor A (VEGFA) gene	Promote the proliferation and migration capacity of OA chondrocytes	Liu et al. (2022b)
			Suppress the apoptosis of OA chondrocytes	
iPSCs-derived exosomes	Osteoarthritic chondrocytes	Unknown	Stimulate the proliferation and migration of chondrocytes	Zhu et al. (2017)
AFSCs-derived exosomes	Osteoarthritic chondrocytes	TGF-β and IDO mediated immunosuppressive	Cartilage repair	Zavatti et al. (2020)
		Promote M2 polarization		
UMSCs-derived exosomes	Osteoarthritic chondrocytes	MiR-100-5p/NOX4 signaling pathway	Alleviate apoptosis	Yan and Wu, (2020)
ASCs-derived exosomes	Human stem cells	Regulation of genes expression by upstream regulatory pathways	Bone and cartilage regeneration	Lei et al. (2022)
PRP-Exos	Osteoarthritic chondrocyte	WNT/β-catenin signaling pathway	Stimulate chondrocytes proliferation and migration	Liu et al. (2019)
			Decrease OA chondrocytes apoptosis	
IPFP-Exos	Osteoarthritic chondrocytes	MiR-100-5p and its downstream pathway mediated downregulation of mTOR.	Reduce articular cartilage destruction	(Wu et al., 2019; Li et al., 2021a)
			Improve gait function	
Chondrocytes-derived exosomes	Osteoarthritic chondrocytes	Promote M2 macrophage infiltration	Restore the metabolism of degenerative chondrocytes	Zheng et al. (2019)
			Modulate immune reactivity	
SFCs-derived exosomes	Osteoarthritic chondrocytes	MiR-126-3p mediated anti-inflammatory signaling	Suppress chondrocyte inflammation and cartilage degradation	Zhou et al. (2021)
		Decrease the production of proinflammatory cytokine		

## Exosomes and cartilage tissue engineering

Currently, osteochondral repair through encapsulating native cells such as chondrocytes and MSCs or growth factors by the injection of polymers generated from natural biomaterials or synthetic materials has been explored. However, there are still many limitations to its clinical application. For example, studies revealed that the autologous chondrocytes may be infective in elderly patients, and the long-lasting healing effects are also restricted by their short half-life property and the poor intracellular delivery of growth factors (Yang et al., 2017; Khorshidi and Karkhaneh, 2018; Chen et al., 2021). Exosomes with the bioactive constituents, including cytokines, growth factors, transcription factors, are secreted from the secreted cells, and are nonliving. Therefore, exosomes do not require *in vitro* maintenance, can easily integrate with target cells, and have been noted as a novel engineering strategy for osteochondral injuries in OA (Bei et al., 2021).

Modified exosomes can be derived from MSCs that are transfected with transcription factors to target different stages and phenotypes of OA. Liu et al. (2018) derived exosomes from lncRNA-KLF3-AS1-treated MSCs with enhanced KLF3-AS1 expression for the treatment of OA. They found that the ECM components Col2a1 and aggrecan were upregulated, whereas important inflammatory markers, such as MMP-13 and RUNX2, were reduced. These findings confirmed that the engineered exosomes facilitated chondrocyte proliferation and alleviated apoptosis.

Based on its soft-like quality, hydroscopicity, elastic mechanical function, and prominent biocompatibility, implantation of hydrogels is currently a major method to load various growth factors and stem cells for local cartilage repair (Taylor and Panhuis, 2016; Zhao et al., 2017; Murphy et al., 2020). To improve the poor adhesion of hydrogels to wet tissue, Zhang et al. (2021a) invented highly adhesive hydrogel combining alginate-dopamine, chondroitin sulfate, and regenerated silk fibroin, which significantly refined the bonding intensity to the wet surface. Relevant experiments based on chemokine signaling pathways and promoting BMSC differentiation into chondrocytes, the novel biomaterial showed therapeutic potential for OA. Hu et al. (2020) creatively combined laponite nanoclay with gelatin methacrylate (Gelma) hydrogel and confirmed that the poor strength and the lack of ordered structure in the cartilage tissue of the hydrogel can be remedied by nanoclay, ensuring a favorable condition for cell proliferation and differentiation. Recently, Shen et al. (2022) also fabricated a silk fibroin (SF) hydrogel that can be injected to support and maintain exosomes derived from hypoxia-pretreated mesenchymal stem cells for cartilage tissue engineering. The experiment revealed that the injectable silk hydrogel carrying articular chondrocytes and hypoxia-pretreated exosomes (H-Exos) was conducive to ameliorating cartilage degeneration as well as facilitating cartilage repair through the miR-205-5p/PTEN/AKT pathway, indicating a new therapeutic target for cartilage tissue engineering utilizing exosomes and articular chondrocytes loaded with SF hydrogel. Nevertheless, more studies are needed to further explore SF/H-Exos therapeutic effects without articular chondrocytes and whether the therapeutic strategy is viable in OA patients.

A series of studies confirmed that exosomes can be adsorbed over the cryogel surface. Nikhil et al. (Nikhil and Kumar, 2022) shed light on the promising role of exosomes combined with chitosan-gelatin-chondroitin (CGC) cryogel extract, which effectively promoted chondrocyte proliferation and migration. Notably, both exosomes and CGC cryogels facilitate chondrocyte proliferation alone and exert more potent positive effects when used in combination. Therefore, exosomes combined with cryogels can be potentially used as an alternative cartilage tissue engineering strategy for OA patients.

**FIGURE 1:**
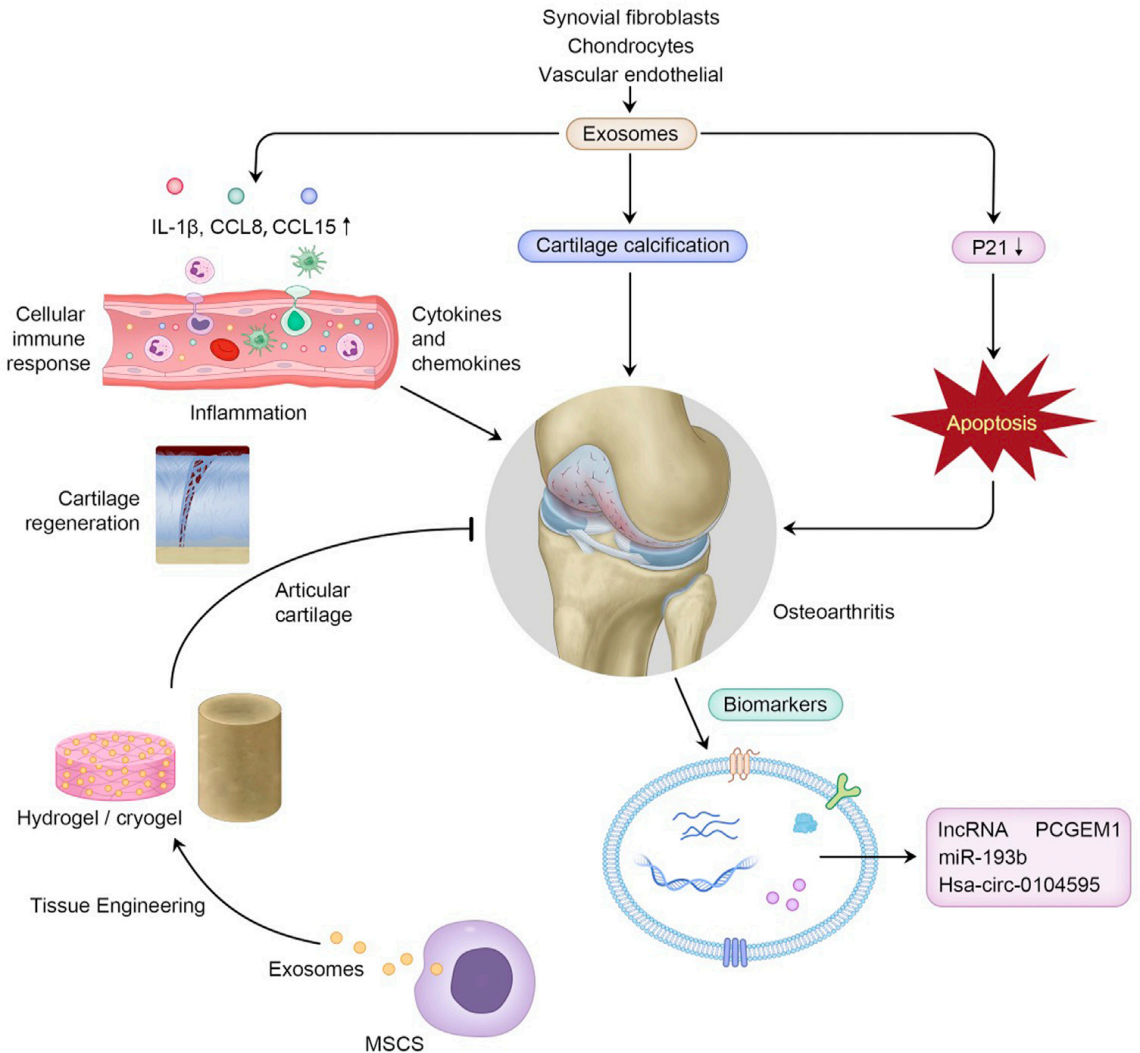
Exosomes in OA pathogenesis, and their clinical potential to serve as biomarkers in the diagnosis of OA and to function in cartilage tissue engineering combined with biomaterials.

In addition to CGC and hydrogels, other biomaterials including titanium nanotubes and a delivery platform combined with exosomes, are also emerging (Wei et al., 2019; Swanson et al., 2020) (Table 3) (Figure 1)

**TABLE 3:** Exosomes and cartilage tissue engineering.

Materials	Exosomes source	Target tissue	Advantages	Efficacy	References
CGC	Articular chondrocytes	Articular cartilage	Porosity, pore size, swelling kinetics and mechanical strength	Enhance proliferation and migration of the chondrocytes	Nikhil and Kumar, (2022)
Gelma/nanoclay/sEVs hydrogel	Human umbilical cord MSCs	Articular cartilage	Water content, thixotropy, desirable biocompatibility and high strength	Promote cartilage regeneration	Zhang et al. (2021a)
Titanium nanotubes	Bone morphogenetic protein 2/macrophage	Bone marrow stromal cell	Excellent biocompatibility and mechanical properties	Promote osteogenic activity and bone regeneration	Wei et al. (2019)
Poly(lactic-co-glycolic acid) and poly (ethylene glycol) triblock co-polymer	Human dental pulp stem cells	Bone marrow stromal cell	Safe degradation rate and safe degradation biproducts	Promote osteogenic differentiation and bone healing	Swanson et al. (2020)

## Conclusion

As pivotal intercellular signaling messengers, exosomes provide an advanced strategy for the assessment of pathological cellular or tissue states in OA. Exosomes in biological fluids, such as synovial fluid and plasma, have shown great potential as novel biomarkers, as they contain specific nucleic acids (miRNAs, lncRNAs), proteins, lipids, amino acids, and metabolites derived from the selected cells and can steadily transfer vesicular substances. RNAs, especially circRNAs, have emerged as novel biomarkers for OA. Moreover, exosomes promote angiogenesis, bone remodeling and chondrocyte proliferation and migration while inhibiting osteoarthritic chondrocyte apoptosis and attenuating cartilage inflammation. Exosomes derived from different types of mesenchymal stem cells, especially BMSCs, AMSCs, SMSCs and EMSCs, and several types of other cells or tissues, including PRP, IPFP, chondrocytes, and SFCs, may ameliorate osteoarthritis as a disease-modifying osteoarthritis cell-free products through the stimulation of cartilage regeneration and inhibition of chondrocyte apoptosis. Although presenting studies reveal that exosome-derived MSCs exert better therapeutic effects, it remains a challenge for us to determine which source exhibits a preferable therapeutic role in OA. In addition, facilitated by biocompatible materials, including hydrogels and cryogels, exosomes exert increasing therapeutic effects by cartilage tissue engineering for OA. In the future, we expect that individualized treatment modalities based on exosomes will represent therapeutic strategy for OA.
